# Enhancing Chronic Non-Cancer Pain Management: A Systematic Review of Mindfulness Therapies and Guided Imagery Interventions

**DOI:** 10.3390/medicina60050686

**Published:** 2024-04-23

**Authors:** Beatriz Manarte Pinto, Isaura Tavares, Daniel Humberto Pozza

**Affiliations:** 1 Experimental Biology Unit, Department of Biomedicine, Faculty of Medicine of Porto, University of Porto, 4200-319 Porto, Portugal; bea.manarte.2@gmail.com (B.M.P.); isatav@med.up.pt (I.T.); 2 Institute for Research and Innovation in Health and IBMC, University of Porto, 4200-135 Porto, Portugal

**Keywords:** mindfulness, imagery, psychotherapy, pain management, pain perception, psychological distress, opioid consumption

## Abstract

*Background and Objectives*: There has been an increasing interest in the use of non-pharmacological approaches for the multidimensional treatment of chronic pain. The aim of this systematic review was to assess the effectiveness of mindfulness-based therapies and Guided Imagery (GI) interventions in managing chronic non-cancer pain and related outcomes. *Materials and Methods*: Searching three electronic databases (Web of Science, PubMed, and Scopus) and following the PRISMA guidelines, a systematic review was performed on Randomized Controlled Trials (RCTs) and pilot RCTs investigating mindfulness or GI interventions in adult patients with chronic non-cancer pain. The Cochrane Risk of Bias Tool was utilized to assess the quality of the evidence, with outcomes encompassing pain intensity, opioid consumption, and non-sensorial dimensions of pain. *Results*: Twenty-six trials met the inclusion criteria, with most of them exhibiting a moderate to high risk of bias. A wide diversity of chronic pain types were under analysis. Amongst the mindfulness interventions, and besides the classical programs, Mindfulness-Oriented Recovery Enhancement (MORE) emerges as an approach that improves interoception. Six trials demonstrated that mindfulness techniques resulted in a significant reduction in pain intensity, and three trials also reported significant outcomes with GI. Evidence supports a significant improvement in non-sensory dimensions of pain in ten trials using mindfulness and in two trials involving GI. Significant effects on opioid consumption were reported in four mindfulness-based trials, whereas one study involving GI found a small effect with that variable. *Conclusions*: This study supports the evidence of benefits of both mindfulness techniques and GI interventions in the management of chronic non-cancer pain. Regarding the various mindfulness interventions, a specific emphasis on the positive results of MORE should be highlighted. Future studies should focus on specific pain types, explore different durations of the mindfulness and GI interventions, and evaluate emotion-related outcomes.

## 1. Introduction

According to the International Association for the Study of Pain (IASP), pain consists of an unpleasant emotional and sensory experience linked to actual or potential tissue damage, driving individuals to seek medical attention [1,2]. Chronic Pain (CP) is considered a pathological condition characterized by its persistence beyond the healing period, typically around 3 months [2,3,4]. The source of the physical pain may or may not be identified [5,6]. CP’s prevalence is very diverse, ranging from 11% to 63% [7,8,9,10].

The biopsychosocial model describes CP as a dynamic interplay of physiological, emotional, and social factors [11], including vulnerability, conditioned responses, and emotional–cognitive states, impairing the quality of life and with an economic impact [12]. In this context, sensory variables are important to assess, namely pain intensity, but also emotional variables, particularly anxiety and depression. More complex aspects are also to be considered, namely life meaning, stress, sleep quality, well-being, opioid consumption, unpleasantness, acceptance, catastrophizing, and interference [13,14,15,16]. This last parameter can be defined as a “construct of the self-reported consequences of pain on activities” and “satisfaction in social relationships with family and friends and enjoyment of participation in work and social activities” [17]. Herein, besides the classical pharmacological approaches, a multidimensional approach of CP should be considered in personalized and integrative pain management [18,19]. Due to analgesics’ side effects [20], the inability to promote pain relief for some CP patients [21], or the invasiveness of low-resolution surgical procedures [7], pain clinics could benefit from the inclusion of Cognitive Behavioral Therapies (CBTs), such as mindfulness approaches, in an effort to reduce suffering.

Mindfulness has been defined as the awareness that arises through paying attention in a particular way: on purpose, in the present moment, and non-judgmentally [22]. This approach empowers CP patients to change their perception of pain, fostering coping skills, reducing suffering, and consequently improving their overall quality of life [23,24,25]. Mindfulness interventions can be used alongside other treatments or even as a stand-alone approach [25] through several approaches, including group-based programs, retreats, comprehensive treatment programs, such as cognitive behavioral stress management and acceptance and commitment therapy, and via internet and smartphone apps [23]. The most currently used programs classically last for 8 weeks and include Mindfulness-Based Stress Reduction (MBSR), Mindfulness-Based Cognitive Therapy (MBCT), and Mindfulness Self Compassion (MSC). Recently, Mindfulness-Based Relapse Prevention (MBRP) and Mindfulness-Oriented Recovery Enhancement (MORE) are also used in specific situations.

MBSR aids patients coping with various challenges through body awareness and acceptance in weekly sessions, a retreat day, and home-based sessions [26,27,28,29,30]. MBCT combines cognitive therapy and psychoeducation to foster the acceptance of unwanted feelings and thoughts, emphasizing metacognitive awareness [27,31,32], which may prevent depression relapses [33,34]. MSC provides tools for better and proactive self-care, allowing for relief from suffering, including CP [35,36,37,38]. MBRP is an intervention to reduce the probability and intensity of relapse by identifying its risk factors and increasing awareness, exposure, and behavioral flexibility in daily cognitive and emotional experiences [39,40,41]. MORE uses social–behavioral learning theory to enhance participant motivation and engagement, and, by combining CBT, psychological concepts, and mindfulness in a group program, it uniquely addresses and improves psychiatric symptoms, physical pain, and addictive behavior [42,43,44,45,46]. Spiritualized Mindfulness (SPM) is a combined mindfulness and spiritualization technique which aims to cultivate a spiritual feeling through framing and explaining the technique, followed by guided meditation focused on the spirituality–breath connection [47]. In spite of the benefits, mindfulness can lead to increased false-memory recall, temporary increases in pain, and agitation or anxiety derived from the increased awareness of bodily sensations in certain patients [48] and, herein, specific features need to be considered, such as individual characteristics, preferences, and medical history.

Another integrative mind–body intervention directed to CP that emerged recently is Guided Imagery (GI) [49,50]. This intervention is different from mindfulness [51,52] as it incorporates techniques such as the generation or recalling of mental images and/or verbal suggestions using, for example, storytelling, drawing, or interpretation of dreams. Thus, the success of this technique depends namely on the therapist leading the patient to achieve the desired response, particularly in pain reduction [53], and on the patient’s focus and relaxation, achieved by availing oneself of techniques, such as diaphragmatic breathing or progressive muscle relaxation [54,55]. GI reduces stress, pain, anxiety, analgesic intake, blood pressure, heart frequency, and fear, while improving sleep, immunity, psychological well-being, and energy [54,56,57,58,59,60,61]. The efficacy of GI remains unclear in regard to pain treatment other than cancer and musculoskeletal pain [50]. Therefore, since this is considered an effective, feasible, safe, and accessible cognitive behavioral tool [53], its benefits in non-cancer CP should be deeply evaluated.

The aim of this systematic review was to assess the effectiveness of mindfulness therapies and GI interventions in managing non-cancer CP and related outcomes. In this review, non-cancer CP was primarily attributed to osteoarticular causes [21,62,63], with the lower back being the most common location of CP [62,64]. By synthesizing the best available evidence, this review aims to provide valuable guidance to healthcare providers and individuals seeking evidence-based interventions for chronic non-cancer pain management.

## 2. Materials and Methods

A systematic review was conducted according to the Preferred Reporting Items for Systematic Reviews and Meta-Analyses (PRISMA) guidelines. The PICO question for this review was “In chronic non-cancer pain subjects (P), does the use of mindfulness therapies and/or GI interventions (I) compared to standard care or other interventions (C) result in improved pain management and related outcomes (O)?”. If yes, which one is better?

A search in three electronic bibliographic databases, which included Web of Science, PubMed, and Scopus, was carried out in October 2022. The search strategy was built up combining search words (Mindfulness, Imagery, Psychotherapy, Chronic, Pain, Chronic Pain) and MeSH terms (Mindfulness; Imagery, Psychotherapy; Chronic Pain). The search strategy used for PubMed was the following: (“Mindfulness” [Mesh] OR “Imagery, Psychotherapy” [Mesh]) AND “Chronic Pain” [Mesh]; for Scopus we used: (Mindfulness OR Imagery) AND Psychotherapy AND chronic AND pain; and for Web of Science the keywords were: “Mindfulness” AND “Imagery” AND “Chronic Pain”. The last strategy was adopted to optimize data extraction from databases.

The inclusion criteria applied comprised: adult human participants diagnosed with chronic non-cancer pain; participants who have completed a mindfulness or GI intervention; measures of pain intensity and/or pain-related outcomes; published in English; a sample size of at least 10 participants per group; Randomized Controlled Trials (RCTs; including pilots if the final study was not published). The exclusion criteria included studies in which participants had cancer-related pain or who had not completed a mindfulness or GI intervention, as well as the duplicated studies, not including measures of pain intensity and/or pain-related outcomes, observational studies, case reports, and case series.

Titles and abstracts were screened by one of the authors to assess their relevance and alignment with the objective of this systematic review. After this initial selection, a full-text review was conducted and information from each selected study was extracted, including the characteristics of the participants and the conclusions drawn. The studies were then systematically evaluated, ensuring that only studies with the appropriate methodology and outcomes were included in the systematic review and that the results were valid and reliable.

Regarding the data synthesis process, the first step involved conducting a thorough extraction of the relevant data from each study, including the participants’ characteristics, number of participants, the intervention details, and the outcome measures. These data were then organized in a chart and subdivided into classical mindfulness interventions, novel mindfulness interventions (studies that used MORE), and in GI studies. Subsequently, a qualitative analysis of the findings was conducted to identify patterns and trends in the data including risk of bias. This consisted in grouping the studies based on their similarities and differences and conducting a critical appraisal of the strengths and limitations of each study by using the Cochrane Risk of Bias Tool [65].

The search, selection, evaluation, and extraction process were then reviewed by the other two authors, and the analysis and categorization of results was performed by all the authors involved in this systematic review. If any discrepancies were present, the solution was found through consensus.

Regarding the degree of agreement between the authors of this review, in the initial analysis made by two of them (BM and DP), the k was 100%. When comparing the studies accepted for inclusion by these authors and the ones accepted by the third author (IT), the k was 97% due to disagreement with one of the trials that was excluded due to the use of both interventions simultaneously (mindfulness and GI).

## 3. Results

**Figure 1:**
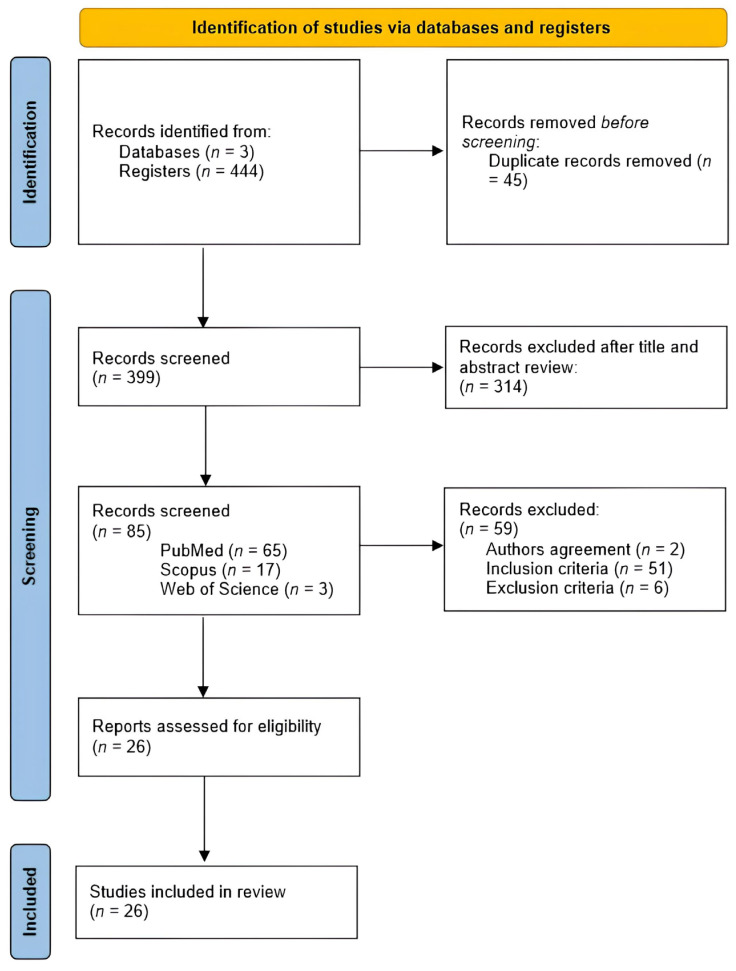
PRISMA flow-chart of literature search and study screening and inclusion.

The PRISMA flow-chart is depicted in Figure 1. The initial electronic search assembled 444 references, of which 45 were removed since they were duplicated records. From the 399 articles remaining, 373 were excluded due to the non-fulfillment of the inclusion criteria and/or due to some of their features being part of the exclusion criteria. Of them, 326 were excluded due to their target population being patients with cancer-related pain or who had not completed a mindfulness or GI intervention or due to lack of inclusion of mindfulness or GI methods; 2 were excluded because the full text was written in German; 45 were excluded since the study design was not consistent with an RCT or a pilot of an RCT.

After the review process was completed, 26 studies were included in this review, 7 pilot RCTs (27%) and 19 RCTs (73%).

Table 1 displays the selected studies. All the trials included various pain-related outcomes: intensity, interference, unpleasantness, acceptance, and catastrophizing. Regarding pain intensity measurement, some studies assessed intensity using a Numeric Rating Scale (NRS), on a scale from 0 (“no pain”) to 10 (“worst pain imaginable”) [47,66,67,68,69,70,71,72,73,74,75,76,77,78,79], by asking the patients the one number that best describes their pain, modifying if they were assessing the average, worst, or current pain. Meanwhile, others utilized a Visual Analogue Scale (VAS) [38,80,81,82], also on a scale from 0 (“no pain”) to 10 (“worst pain”). Additionally, some studies used an NRS but used different scales. Cooperman et al. [83] made this assessment with subscales of the RAND 36-Item Short Form Health Survey, with the scores for each factor ranging from 0 to 100, where higher scores indicated less pain. Lewandowski et al. [84] used the Wong–Baker FACES scale to evaluate pain intensity at baseline but employed another method to assess this parameter throughout the study, which utilized descriptor word groups. Baird et al. [85] used the pain scale from the Arthritis Impact Measures (AIMS2) to assess this. None of the studies defined the interpretation of the intensity as low, moderate, or high.

Furthermore, most of them also focused on some emotional variables, namely depression, anxiety, meaning in life, stress, and sleep disturbances, as well as quality of life.

In terms of interventions, four trials used MORE [70,71,72,83], considered as the novel mindfulness intervention, eighteen trials used more classical mindfulness interventions (two Mindfulness Meditation (MM) [76,78], six MBSR [66,67,74,77,80,86], one MBCT [68], one MSC [38], one Mindfulness in Action (MIA) [69], one mindfulness-based pain management program [88], one SPM [47], one online mindfulness intervention [73], two non-specified mindfulness-based therapies [75,87], and two other mindfulness approaches [79,82]), and four trials used GI [81,84,85,89].

The participating adult populations were from a great variety of origin countries (50% from the USA [47,66,67,70,71,72,78,79,82,83,84,85,86], 15.4% from the United Kingdom [73,75,77,88], 7.7% from Spain [38,81], 3,8% from Sweden [74], 7.7% from Australia [68,87], 3.8% from the United Arab Emirates [76], 3.8% from Denmark [80], 3.8% from Ireland [69], and 3.8% from Germany [89]), with 38.3% of them being European countries, and included a pronounced variety of CP types. A total of 2964 patients (2762 in mindfulness trials and 202 in GI interventions) were included in the analyzed studies. The studies with the smallest sample have 28 patients [85,88], and the study with the largest sample has 342 patients [80]. The duration of the CP was more than 8 years in 11 trials [66,68,70,71,73,74,75,76,79,80,84,86] and less than 8 years, but more than 3 months, in 9 trials [38,67,69,77,78,81,82,83,89], while 6 trials did not report the duration of pain [47,72,85,87,88].

Regarding study results, in all four of the MORE trials [70,71,72,83], the intervention was significantly better at improving pain intensity and opioid consumption and/or craving than control. One of them reported an improvement in pain interference and in stress levels [72], one described a significant reduction in anxiety and depression levels [83], and one reported a significant improvement in both pain interference and depression [70] and one in positive affect, meaning in life, and savoring [71].

The trials that focused on MM showed that this intervention led to a significant reduction in pain intensity [76,78]. In the Williams, Day et al. trial [78], MM did not significantly reduce anxiety and depression, but it was able to reduce pain interference more than the other interventions.

Day, Ward et al. [68] focused on MBCT and showed a significant decrease in pain intensity, although it was not different from the other groups. They also demonstrated a significant reduction in pain interference and depression levels compared with the other interventions, as well as a significant difference in opioid use between the pre-treatment phase and the 3-month follow-up period.

Out of six trials that studied the effects of MBSR, two of them showed that MBSR led to a significant decrease in pain intensity [66,74], while four verified a pain reduction that did not differ from the control group [67,77,80,86]. Burns, Jensen et al. [66] showed that MBSR led to a more pronounced reduction in pain interference, depressive symptoms, and sleep disturbance than the control. Henriksson et al. [74] showed a small effect size of MBSR on pain interference, although better than the control, and a significant decrease in affective distress and pain acceptance than control. In the Morone et al. trial [86], MBSR showed a more pronounced decrease in catastrophizing than control but not in depression. In Cherkin’s study [67], MBSR significantly improved pain bothersomeness and anxiety and depression levels, although CBT demonstrated better results in these last two variables. The results of la Cour and Petersen [80] showed a significant improvement in anxiety and pain acceptance than the control, which was not verified in catastrophizing and depression. In the Ussher et al. trial [74], MBSR led to a significant reduction in distress and in pain interference in the clinic setting when compared with control.

With respect to the remaining trials of mindfulness interventions, two did not assess the effects of the intervention on pain intensity [47,88], one showed a significant decrease in this variable with the respective intervention [79], and the other six did not show differences between groups for the same variable [38,69,73,75,82,87]. In the Zgierska et al. trial [79], the intervention led to a significant increase in pain acceptance, but no better than control, which also happened regarding mindful attention, perceived stress, and opioid dosage used. In the Torrijos-Zarcero et al. study [38], MSC led to a significant decrease in anxiety levels, pain interference, and depressive symptoms, and a higher increase in pain acceptance, in comparison to CBT. MSC significantly reduced pain catastrophizing. Polaski et al. [82] showed that the Meditation and Exercise Trial (MedExT) did not lead to any significant improvement in anxiety levels. In the Hearn and Finlay trial [73], the mindfulness online intervention led to a significant improvement in anxiety levels and pain catastrophizing, with this effect on pain unpleasantness only in T2 level lesions. Dowd et al. [69] showed that MIA significantly improved catastrophizing, pain acceptance, and pain interference but not anxiety and depression levels. The Howarth et al. trial [75] did not demonstrate significant differences in anxiety and depression levels, Cathcart et al. [87] did not assess emotional variables after treatment. Brown and Jones [88] showed a statistically significant improvement in mental health and in affective clinical pain score with a mindfulness-based pain management program.

Regarding the GI studies, two of them reported a significant reduction in pain intensity [81,85]. In another study, since the variables evaluated were different, pain intensity was not assessed directly, but in the intervention group pain became changeable and there was no recurrence of constant pain [84]. Furthermore, the Alexander Technique (AT) demonstrated a better efficacy in improving pain intensity and satisfaction levels than GI [89]. In the Onieva-Zafra et al. study [81] there was a better improvement in depression levels with GI than with control. Two trials [84,85] did not evaluate emotional variables.

**Figure 2:**
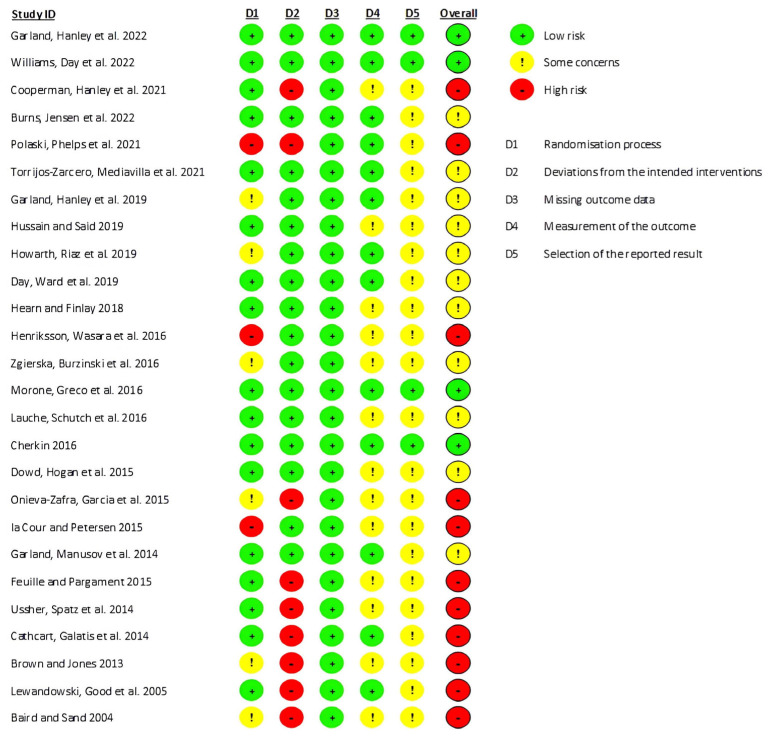
Evidence quality assessment of the studies included in this review using the Cochrane Risk of Bias Tool. The risk of bias is represented in five categories and with its overall score for each study. A code of colors is used: green-low risk of bias; yellow-some concerns about bias; red-high risk of bias [38,47,66,67,68,69,70,71,72,73,74,75,76,77,78,79,80,81,82,83,84,85,86,87,88,89].

Concerning the methodological quality, eleven studies were classified as having a “high risk” of bias, eleven as “moderate risk”, and four as “low risk”. A great percentage of this risk arose from the impossibility of blinding the participants and the investigators due to the nature of the interventions applied (Figure 2).

## 4. Discussion

### 4.1. General Findings

To the best of our knowledge, this is the first systematic review addressing mindfulness and GI interventions in chronic non-malignant pain. Overall, mindfulness-based interventions demonstrated a more useful role in improving emotional outcomes than pain intensity, while GI interventions were found to be useful in reducing pain intensity. Since only two GI-focused studies [81,89] addressed their impact on emotion outcomes, we cannot definitely state whether these interventions may have a beneficial effect on those variables. However, those two trials showed potential in aiding patients at an emotional level.

The studies analyzed in this systematic review were highly variable across multiple factors, namely in the pain type and duration of the intervention. Regarding the duration of the intervention, it should be noted that there is currently consideration for shortening of the 8-week period of classical MBSR or MBCT programs. This is because the same intervention type may potentially improve pain-related outcomes within a shorter timeframe. This was mainly evident when comparing the effects on emotional variables between the MBSR trials with an 8-week protocol [66,67,74,80,86] and the brief MBSR intervention [77]. The findings of a recent trial [90] indicated that varying durations of MM sessions, whether 10 min or 30 min over a period of two weeks, did not significantly influence the impact on mental well-being. Although this investigation only focused on one mindfulness intervention, and the target population was healthy, it may reinforce the evidence seen in this review. This is interesting as the duration of the interventions was previously viewed as a limitation of the classical mindfulness programs.

All trials focusing on a novel mindfulness intervention demonstrated improvements compared to the control in at least one of the components studied [70,71,72,83]. Regarding the trials that focused on classical mindfulness interventions, five showed no significant difference in the effects of intervention compared to the control group [69,75,78,82,87]. This might happen because three of those trials are pilots and may have had a lower number of participants than the one needed to achieve significant results [75,82,87]. In the MM trial [78], this could be attributed to the fact that the control employed is a hypnosis technique, which has shown similar or better results compared to MM in other trials [91,92]. Regarding MIA [69], the lack of significance in results could arise from the similar benefits it shares with psychoeducation programs, since another trial that studied a similar online treatment demonstrated significant differences in pain-related outcomes when compared with a waiting list control, although it also exhibits a higher rate of adherence [93]. Since MIA was used in a diverse group of patients with varying types of pain, there could have been variable efficacy across these different pain types. MIA could have been effective in one type of pain, but ineffective in another, which could eventually lead to a non-significant result compared to the control.

On the other hand, thirteen studies showed that mindfulness was better than the control in at least one of the variables studied [38,47,66,67,68,73,74,76,77,79,80,86,88].

Regarding the GI studies, three of them reported positive outcomes in several of the variables under investigation [81,84,85]. In the study that compared the AT with GI, the intervention did not achieve results as good as those observed with AT [89].

### 4.2. Effects on Pain Intensity

In all MORE trials there was a significant reduction in pain intensity when compared with supportive group psychotherapy or treatment as usual [70,71,72,83]. The studies on classical mindfulness reported a significant decrease in pain intensity for MM alone [76] or combined with cognitive behavior [79]. However, in two other MM trials, this effect on pain did not happen [68,78]. This may be related to the control being either hypnosis [78] or MBCT [68], that may be equivalent to MM. Due to similarities, the observed outcomes do not appear to be attributable to treatment duration, pain type, or the specific population under study.

Patients undergoing MBSR reported decreased pain intensity compared to the control in only one trial [74], whereas in other five trials this was not observed [66,67,77,80,86]. These differences may derive from the different procedures used in the trial that demonstrated significant results that used audio files and 10 min mindfulness exercises. Although this may appear promising, since there is only one trial using this procedure, there is sparse evidence to conclude about the true value for CP intensity. In general, MBSR does not seem to lead to a significant improvement of pain intensity. Therefore, care must be taken when using it with the main purpose of improving this aspect.

MSC [38] demonstrated a significant ability to improve pain levels. However, not all mindfulness techniques yield the same results. There were no significant differences observed in trials that studied MedExT for treating chronic low back pain [82], as well as for online mindfulness intervention [73], MIA [69], an unspecified mindfulness-based intervention [75], and mindfulness-based interventions with unspecified details [87].

It is important to notice that, although mindfulness techniques were developed with the intent of reducing pain intensity, it has been demonstrated that they are more effective in improving non-sensorial dimensions of pain. Additionally, while mindfulness can have an important role in CP management, improving quality of life by enhancing pain acceptance, its effectiveness may vary from person to person, and its efficacy is dependent on the specific treatment approach. In this context, MORE seems to be the most suitable to reduce pain perception, while other techniques should be chosen carefully when pain reduction is the primary goal. MSC interventions had promising results, but further studies are needed to better evaluate its effects on pain.

A significant reduction in pain was reported with GI alone, either with 4 days of treatment [84] or over a 4-week period [81], indicating a short-term benefit that can be extended according to patients’ needs. The combination of GI and Progressive Muscle Relaxation (PMR) was also found to be beneficial when compared with a control group [85]. However, when compared with the AT, GI was found to be inferior regarding pain intensity [89]. This also suggests that the employment of GI-based methods can lead to a significant decrease in CP perception and the treatment choice should be based on patients’ needs, as happens with other mind–body interventions.

### 4.3. Effects on Non-Sensorial Dimensions of Pain

Although quality of life was not assessed in MORE studies, three of them demonstrated a significant reduction in depression and/or anxiety/stress levels [70,72,83], two trials showed a reduction in pain interference [70,72] and one study reported a significant improvement in positive affect [71]. This demonstrated that MORE is an important therapy to approach the complex and multidimensional nature of pain and related emotional variables, and, therefore, should be particularly considered for CP patients experiencing effects on their mental well-being. These results are in accordance with a previous meta-analysis on CP, along with addictive behavior and psychiatric distress [46].

MM did not demonstrate superiority to hypnosis [78] nor to MBCT [68] in improving pain interference, anxiety, and depression. However, when administered alongside CBT [79], mindfulness led to a significant improvement in pain acceptance, aligning with the objectives of this therapeutic approach. Three of four MBSR trials demonstrated significant improvements in quality of life directly [67,74,80]. Furthermore, MBSR led to a significant improvement in pain-related outcomes, such as pain interference, pain acceptance, and pain catastrophizing and a significant reduction in anxiety/distress levels [67,74,77,80]. MSC managed to significantly decrease anxiety and depression levels, as well as reduce pain interference and catastrophizing and increase pain acceptance [38].

No significant improvements in quality of life [83], anxiety/stress, and depression were observed in the trials with a non-specified mindfulness intervention when compared with the control [75,87]. It is important to note that patients should receive the most appropriate and personalized treatment to achieve better results, especially when managing such a complex problem as CP. Sometimes, relying solely on one approach may not be sufficient, and patients may lose confidence in mind–body therapies. This situation can be exacerbated if the patient has already experienced failed treatments, including pharmacological ones. Consequently, not only may emotional variables related to pain fail to improve but anxiety, depression, catastrophizing, and sleep disturbances could worsen, further intensifying the experience of pain. In this context, it becomes exceedingly important to select the appropriate treatment from the beginning.

Mindfulness interventions were typically conducted in person. However, advancements in new technologies have now enabled alternative approaches, allowing for greater flexibility and accessibility. Virtual platforms and mobile applications, for instance, have revolutionized the way mindfulness practices can be delivered, reaching a wider audience and accommodating various schedules. This shift towards technology-mediated interventions has expanded the possibilities for individuals to engage in mindfulness training from the comfort of their own environments, promoting convenience and potentially enhancing the integration of mindfulness into their daily lives. Furthermore, online mindfulness also allows a significant reduction in depression levels and severity, as well as in pain unpleasantness and catastrophizing, although it did not demonstrate a significant effect on quality of life [73]. Again, caution must be taken when opting for an online approach in the complexity of CP. Ideally, patients should be initially seen in person for a comprehensive first evaluation of their current situation. Subsequently, follow-up therapy can be conducted conveniently online for those for whom it is the only feasible way to continue treatment.

Selecting the most suitable mind–body therapy for each patient is crucial, as not all approaches will effectively address the concerns voiced by individuals. For instance, a therapy that focuses on relaxation might be more beneficial for someone experiencing stress-related issues, while another individual with CP might find greater relief through techniques emphasizing pain management and physical rehabilitation. Tailoring the choice of therapy to the specific needs and preferences of each patient enhances the likelihood of positive outcomes and contributes to a more personalized and effective healthcare approach. In this context, MIA was not able to significantly impact pain acceptance, interference, and catastrophizing nor anxiety and depression levels compared with psychoeducation [69]. The reason may also lie in different uses of evidence-based protocols; instead, audio-visual treatment was used.

Although MedExT proved to be useful in reducing low back pain intensity and unpleasantness, this modality did not improve anxiety levels [82]. This could be attributed to the intervention’s potential to offer greater benefits in reducing anxiety levels when patients initially have higher baseline anxiety compared to those who participated in the trial [82]. Furthermore, the lack of a significant result may be due to the limited number of participants, which may have not provided enough statistical power to detect differences that might actually exist.

STM was able to significantly reduce pain-related stress [47]. Although the number of trials directly assessing quality of life were not substantial, those that did suggest that these interventions can inherently benefit this component. Moreover, it seems that they can have a significant positive impact on pain-related outcomes as well as mental health.

Only two GI interventions analyzed emotional variables [81,89]. One showed improvement in satisfaction levels [89] and the other demonstrated a significant amelioration in depression when compared with the control [81]. Since only two of these trials targeted emotional variables, it is challenging to draw definitive conclusions regarding the impact of these interventions on emotions. GI is a relatively less comprehensive therapy, and the multidimensional and complex disease of CP may also require a more holistic mind–body approach. A MORE intervention, for example, which encompasses a wider range of techniques, from mindfulness practices to reappraisal and savoring skills, could be more suitable in addressing the various dimensions of CP. On the other hand, GI’s focus is based on imagery techniques which, although diversified, cannot reach as far as mindfulness when approaching the various pain dimensions.

### 4.4. Opioid Consumption

Understanding opioid consumption is of paramount importance due to its consequences on public health, society, and economy. Opioid abuse not only poses significant risks to individual health but also contributes to societal issues such as addiction, overdose deaths, and economic burdens. Regarding MORE interventions, they were able to significantly reduce craving and/or consumption of opioids in all four trials included in our results [66,67,68,69]. The trials of classical mindfulness interventions did not demonstrate any significant difference in this outcome when compared with the control [68,78,79]. Given this, there is a potential of MORE in improving variables linked to opioid consumption. However, more studies focused on classical mindfulness treatments are required to better evaluate their effects on this important outcome.

### 4.5. Cancer Pain

Ultimately, extensive research has been conducted regarding mind–body approaches in the context of cancer-related pain. A comprehensive systematic review and meta-analysis [94], encompassing mindfulness interventions alongside GI interventions, unveiled noteworthy findings. Notably, a discernible and favorable influence on this particular outcome was observed. More specifically, mindfulness interventions led to significant results in favor of the intervention, while GI interventions showed only a small or even nil effect in favor. These results in cancer pain are similar to those found in our study on chronic non-cancer pain, but the quality of the evidence garnered remains limited and a substantial degree of heterogeneity within the studies was evident. In another systematic review and meta-analysis, there were also moderate to large effect size improvements in pain and opioid-related outcomes with mindfulness but not with GI [95]. Bearing in mind these results and the ones analyzed in this review, most evidence points to a lack of effect of GI in opioid-related outcomes. However, this reinforces the important role of mindfulness in improving these outcomes and therefore it gives a better and greater opportunity in life to these patients.

### 4.6. Limitations

This review has some limitations. Although the type of study included has some of the highest levels of evidence, the sole use of RCTs could lead to the loss of some crucial information on the topic studied. The bias evaluation showed that most studies included have a high or moderate risk of bias, mostly due to lack of allocation concealment, which, given the interventions studied, is often impractical to implement, and blind assessment of outcomes. The latter is a characteristic that could be improved in future trials. Additionally, given the low number of RCTs with intervention groups exposed to GI, it was not possible to exclude trials that did not consider emotional variables, an essential component to study regarding integrative therapies. Furthermore, the heterogeneity of the target population and of the duration of techniques (especially between mindfulness interventions and GI ones) is moderate/high. Finally, a meta-analysis was not possible due to limited eligible studies, high methodological heterogeneity, and a substantial risk of bias in the available research. These factors hindered the reliable aggregation of data and the generation of meaningful conclusions through meta-analytic techniques.

### 4.7. Strengths

This review demonstrates several strengths in its methodology. It exhibits robustness through well-defined inclusion and exclusion criteria, systematic adherence to PRISMA guidelines in the literature search, and rigorous validation of study selection and data extraction by multiple reviewers. Additionally, the review employs the Cochrane Risk of Bias Tool for quality assessment, furnishes comprehensive study and patient characteristics, and ensures a conflict-of-interest-free evaluation by all reviewers.

### 4.8. Future Research

It will be important for further studies to increase their focus not only on the influence of these interventions on pain-related outcomes but also on other dimensions of pain, such as the emotional variables related to it, especially with GI. Given the well-known close relationship between emotions and pain [96,97,98,99], exploring these aspects could provide valuable insights into the effectiveness of interventions like GI. Future studies in CP management should explore the integration of GI with other therapeutic approaches to ensure a comprehensive approach to managing the multidimensional nature of pain. Targeting these components in the future may help obtain to a better understanding of the true potential of mindfulness and GI in the control of CP.

As stated in the review from Eric Garland et al. [95], there should be an effort to at least blind assessors and/or investigators in order to decrease the risk of bias in these types of studies. Since the blinding of participants is very difficult to achieve with mind–body interventions, alternative methods of minimizing bias, such as blinded assessment of outcomes, should be considered in future studies.

It will also be important to study each technique with different durations to assess if it is possible to achieve similar or comparable results in a shorter period, without including subsequent work-at-home sessions. This could make it easier and comfortable for patients to adhere to these techniques. Focusing on more homogenous groups of patients regarding the type of pain and its duration and comparing a classic mindfulness intervention with a GI intervention could provide relevant information about which technique is more effective for chronic non-malignant pain.

## 5. Conclusions

In conclusion, this systematic review underscores that both mindfulness therapies and GI interventions demonstrate promise in managing chronic non-cancer pain and related outcomes. GI interventions exhibited significant reductions mainly in pain intensity. Notably, mindfulness additionally demonstrated significant improvements in pain intensity and emotional variables such as depression and anxiety/stress levels. This, and a beneficial effect in opioid consumption, are especially evident when mindfulness is incorporated with the MORE technique, emerging as a superior choice, offering valuable insights for healthcare providers and individuals seeking evidence-based interventions for chronic non-cancer pain management. Overall, integrating these interventions into chronic non-cancer pain management strategies could offer valuable benefits, but individualized treatment approaches tailored to patient needs and preferences remain important.

## Figures and Tables

**Table 1:** Concise overview of findings from randomized controlled clinical trials employing mind–body therapies on chronic non-cancer pain.

Reference/Study Type	Study/Participant Characteristics	Pain Variables/Emotional State at Baseline	Intervention (*n*)/Time	Control (*n*)/Time	Main Results
Classical Mindfulness Interventions					
[78]/RCT	- Mean age and percentage of females, respectively: - ED: 53.5 ± 13.5; 27% - HYP: 51.0 ± 12.6; 26% - MM: 55.0 ± 13.0; 26% - Hospital - Veterans - Country: USA	- Neuropathic and non-neuropathic chronic pain - >3 months - Intensity of around 6 (NRS) - Anxiety, depression, pain interference, and sleep disturbances - 19–35% taking opioids	HYP (110) - 8 to 10 weeks MM (108) - 8 to 10 weeks	ED (110) - 8 to 10 weeks	- **Intensity**: mean pain intensity reduced with no significant differences between groups. HYP led to greater reductions than ED after 3 months. HYP and MM led to greater reductions than ED after 6 months. - **Emotional variables**: HYP achieved better results for pain interference and for depressive symptoms. Anxiety reduction with no significant difference in the 3 groups. - **Opioid consumption**: no differences.
[76]/RCT	- 100% female and mean age 62.9 ± 12 - Diabetic clinic - Diabetic elderly females - Country: the United Arab Emirates	- Chronic pain associated with diabetes - Between 8 and 11 years of pain - Intensity of 5 points (NRS) - No emotional variables reported	MM (36) - 8 weeks PM (37) - 8 weeks	CM (32) - 8 weeks	- **Intensity**: MM and PM: significant reduction in average daily pain when compared to baseline. CM: non-significant reduction in pain.
[68]/RCT	- 50% female and mean age 51 - University - Country: Australia	- CLBP: spinal pain, arthritis pain, pain from injury, soft tissue or muscle pain, and neuropathic pain - Around 14 years of pain - Intensity of around 4.5/5 points (NRS) - Pain interference and depression - 48% used opioids in the past week	MBCT (23) - 8 weeks	MM (23) - 8 weeks CT (23) - 8 weeks	- **Intensity**: there was a significant improvement in all the groups, with no difference between them. - **Emotional variables**: MBCT showed greater improvement in pain interference and higher depression scores compared to MM, while CT did not differ significantly from MM or MBCT in both aspects. - **Opioid consumption**: No significant difference in opioid use was observed among participants at pre-treatment (48%), post-treatment (43%), and 6-month follow-up (33%). However, there was a significant reduction in opioid use at the 3-month follow-up (28%).
[79]/RCT (pilot)	- >50% female and mean age 51.8 ± 9.7 - Outpatient care - Chronic opioid use - Country: USA	- CLBP - Around 14 years of pain - Intensity of around 6 points (NRS) - Perceived stress and pain acceptance - At least 30 mg/day of morphine-equivalent dose (MED) for at least 3 months	Meditation-CBT + TAU (21) - 8 weeks	TAU (14) - 8 weeks	- **Intensity**: meditation-CBT led to a significant improvement in pain intensity when compared with TAU. - **Emotional variables**: pain acceptance was better in meditation-CBT at 26 weeks. There were no significant differences between groups in pain acceptance, mindful attention, and perceived stress. - **Opioid consumption**: no significant differences in dose of morphine used.
[38]/RCT	- 87.8% female - Public general hospital - Users of a chronic pain liaison program - Country: Spain	- Chronic pain (unspecified) - Most of the patients had >3 years of pain. - Intensity of 7.5 points (VAS) - Catastrophizing, anxiety and depressive symptoms, and pain acceptance - Opioid use not reported	MSC (62) - 8 weeks	CBT (61) - 8 weeks	- **Intensity**: no significant differences found between groups. - **Emotional variables**: MSC led to a significantly greater decrease in anxiety levels, pain interference, and depressive symptoms and a greater increase in pain acceptance in comparison to CBT. MSC significantly reduced pain catastrophizing.
[66]/RCT	- 57.9% female and mean age 52.88 ± 12.16 - University and 3 clinics - Country: USA	- Chronic low back/legs pain - Around 15 years of pain - Intensity of around 5 (NRS) - Depressive symptoms and sleep disturbances - 25% taking opioids	BT (77) - 8 weeks CT (71) - 8 weeks MBSR (67) - 8 weeks	TAU (77) - 8 weeks	- **Intensity**: all 4 groups significantly reduced the pain intensity. BT, CT, and MBSR showed a better reduction than TAU. No differences at 6-month follow-up. - **Emotional variables**: pain interference, depression, and sleep disturbance with higher reductions in BT, CT, and MBSR than TAU. At 6 months, only MBSR had a significant reduction in pain interference and no differences for depression and sleep disturbance.
[74]/RCT	- 93% female and mean age 51.0 - Primary care settings, pain clinic, and online - Country: Sweden	- Chronic pain (unspecified) - Around 14.5 years of pain - Intensity of 6.5 points (NRS) - Pain interference/suffering, affective distress, anxiety, depression, and pain acceptance	MBSR (55) - 8 weeks	Anonymous discussion forum (52) - 8 weeks	- **Intensity**: MBSR with higher pain intensity reduction and larger effect size than control (small effect size). - **Emotional variables**: MBSR with better results in pain interference/suffering, affective distress, pain acceptance, and life satisfaction.
[86]/RCT	- Mean age and percentage of females, respectively: - MBSR: 75 ± 7.2; 66.4% - Control: 74 ± 6.0; 66.2% - Metropolitan Pittsburgh - Community-dwelling older adults - Country: USA	- CLBP - Around 11.5 years of pain - Intensity of 10.5/11 points (NRS scale from 0–20) - Catastrophizing and depression	MBSR (140) - 8 weeks	Health education program (142) - 8 weeks	- **Intensity**: MBSR—significant improvement in current and most severe pain intensity. Average pain intensity did not differ significantly between groups. - **Emotional variables**: MBSR had a greater decrease in catastrophizing and no improvement in depression; changes in quality of life did not reach clinical significance.
[67]/RCT	- 66% female and mean age 49 - Integrated healthcare system - Country: USA	- Non-specific CLBP - More than 3 months - Intensity of around 6 points (NRS) - Pain bothersomeness, pain interference, depressive symptoms, and anxiety - 11.1% using opioids	MBSR (116) - 8 weeks CBT (113) - 8 weeks	UC (113) - 8 weeks	- **Intensity**: MBSR and CBT led to a significantly greater reduction in pain intensity than UC (no statistical differences). - **Emotional variables**: pain bothersomeness (MBSR 44%, UC 27%, CBT 45%; P = 0.01). MBSR was better than UC on the depression and QoL at 8 weeks. CBT led to a greater improvement than MBSR on depression at 8 weeks and anxiety at 26 weeks, and then UC at 8 and 26 weeks on anxiety, depression, and QoL.
[80]/RCT	- Mean age and percentage of females, respectively: - MBSR: 46.52 ± 12.42; 83% - Control: 48.84 ± 12.20; 87% - Multidisciplinary pain center - Country: Denmark	- Non-specific chronic pain: head, cervical, shoulder, arms, thorax, abdominal, low back, legs, pelvis, and anal/genital - 11.8 years of pain in the waiting list group and 7.8 years of pain in the treatment group - Intensity at baseline not reported - Catastrophizing, anxiety, depression, and pain acceptance - Around 6 years of opioid use in the waiting list group and around 5 years in the treatment group	MBSR (43) - 8 weeks	TAU (47) - 8 weeks	- **Intensity**: no significant difference in pain improvement between MBSR and TAU. - **Emotional variables**: MBSR significantly better in improving anxiety levels, mental quality of life, and pain acceptance than TAU. Catastrophizing and depression had no significant difference in improvement.
[77]/RCT	- Mean age and percentage of females, respectively: - MBSR: 59.5 ± 15.8; 85% - Control: 61.6 ± 17.4 ±; 71% - Outpatient pain clinic - Country: UK	- Chronic pain: 80% with back pain (remaining types unspecified) - Around 7 years of pain in MBSR and 5 years of pain in the control group - Intensity of 6 points (NRS) in the treatment group and 5 points (NRS) in the control group - Pain interference, distress, pain acceptance, depression, and anxiety - 56% used opioids in the treatment group and 39% in the control group	MBSR (27) - 24 h (clinic setting and participant’s own environment)	Reading about natural history (28) - 24 h (clinic setting and participant’s own environment)	- **Intensity**: significant reduction in pain intensity with no differences between groups. - **Emotional variables**: MBSR showed a significant reduction in distress and pain interference. Control had a significant reduction in distress. Significant reduction for pain-related distress and for pain interfering with social relations in MBSR when compared with control.
[82]/RCT (pilot)	- 65.8% female and mean age 37.6 ± 15.4 - Clinic - Country: USA	- Chronic low back pain at any severity - More than 6 months - Intensity of around 3 (VAS) - Severe anxiety - Opioids	MedExt (18) - 4 weeks	Audiobook (20) - 4 weeks	- **Intensity**: no significant differences between groups. - **Emotional variables**: no significant improvement in anxiety for both groups.
[73]/RCT (pilot)	- 54% female and mean age 44.4 - The National Spinal Injuries Centre and a hospital - Paraplegia or tetraplegia - Country: UK	- SCI-related pain - From 1 to more than 15 years of pain - Intensity between 5 and 7 points (NRS) - Catastrophizing, depressive symptoms, anxiety, and pain unpleasantness	Online mindfulness intervention (36) - 8 weeks	Internet-delivered psychoeducation (31) - 8 weeks	- **Intensity**: no significant group differences. - **Emotional variables**: mindfulness led to a greater reduction of depression severity, anxiety levels, pain unpleasantness, and pain catastrophizing compared with psychoeducation. No significant differences in quality of life levels.
[69]/RCT	- 90.3% female and mean age 44.53 ± 12.25 - National university - Country: Ireland	- Chronic non-cancer pain: upper and lower back, leg and knee, neck, hand/arm/wrist, head, abdomen, shoulder, muscles, chest, hip, and others - More than 6 months - Intensity of around 6 points (NRS) - Catastrophizing, anxiety, depression, pain interference, and pain acceptance	MIA (62) - 6 weeks	PE (62) - 6 weeks	- **Intensity**: no significant differences between and within groups. - **Emotional variables**: catastrophizing, pain acceptance, and pain interference improved significantly in both groups, but no significant difference between each other; there was no significant differences between and within the groups for anxiety and depression levels.
[47]/RCT	- 82% female and mean age 19.9 ± 3.5 - A Midwestern university campus and in the local community - Migraineur adults - Country: USA	- Chronic migraine - Around 6 migraines per month - Intensity of around 6 points (NRS) - Stress	STM (22) - 2 weeks SPM (27) - 2 weeks	R (25) - 2 weeks	- **Intensity**: not reported. - **Emotional variables**: more significantly reduced pain-related stress in STM than in R.
[75]/RCT (pilot)	- Mean age and percentage of females, respectively: - MBI: 54.7 ± 12.5; 65% - Control: 52.8 ± 12.2; 62% - Physiotherapy and pain clinics - Country: UK	- Chronic pain: 56% with back pain (remaining types unspecified) - Between 8 and 11 years of pain - Intensity of around 5.5 points (NRS) - Catastrophizing, anxiety, and depression	MBI (37) - 4 weeks	Distraction audios (34) - 4 weeks	- **Intensity**: no significant differences between groups. - **Emotional variables**: no significant differences between groups in anxiety, depression, and quality of life, but there was a significant improvement in coping with MBI.
[87]/RCT (pilot)	- Mean age and percentage of females, respectively: - SAIPAN: 45.78 ± 13.10; 57% - Control: 45.26 ± 14.18; 68.5% - University - Not receiving treatment for headache - Country: Australia	- CTH - Frequency of 10 to 11 days in a fortnight - Intensity of around 2/2.5 points (NRS scale from 0 to 5) - Anxiety, depression, and stress	MBT (23) - 3 weeks	Waiting list control (19) - 3 weeks	- **Intensity**: no significative differences between and within groups. - **Emotional variables**: no change in stress, depression, or anxiety in both groups.
[88]/RCT	- Mean age and percentage of females, respectively: - MBPM: 48 ± 10; 80% - Control: 45 ± 12; 69% - Open study - Musculoskeletal pain - Country: UK	- Chronic pain: fibromyalgia, rheumatoid arthritis, cervical nerve root impingement, osteoarthritis, psoriatic arthritis, low back pain, and ankylosing spondylitis - Pain duration not measured - Pain intensity not measured - Pain unpleasantness and affective pain	MBPM (15) - 8 weeks	TAU (13) - 8 weeks	- **Intensity**: not assessed. - **Emotional variables**: MBPM led to a statistically significant improvement in mental health and significant reduction of affective clinical pain score but not in comparison to TAU.
**Novel Mindfulness Interventions**					
[70]/RCT	- 63.6% female, mean age 51.8 ± 11.9 - Primary care clinics - Misusing opioid therapy - Country: USA	- Chronic pain: osteoarthritis, fibromyalgia, neuropathic, cervical, back, pelvic and extremity pain, headache, and irritable bowel syndrome. - Around 15 years of pain - Intensity of around 5 (NRS) - Most patients had major depression and opioid use disorder - Taking opioids for at least 90 days	MORE (129) - 8 weeks	SG (121) - 8 weeks	- **Intensity**: significantly lower after MORE than after SG (50% vs. 29.3% at 9 months). - **Emotional variables**: significant reduction in pain interference with MORE than with SG (58.6% vs. 25.3% at 9 months), mostly due to a reduction in depression subscale scores. - **Opioid consumption**: less consumption of opioids with MORE than with SG (45% vs. 24.2%) and greater dose reduction with MORE (35.5%) vs. SG (15.9%).
[83]/RCT (pilot)	- 50% female and mean age 50.4 - 2 clinics - Methadone for opioid use disorder - Country: USA	- Non-malignant chronic pain: lower back, arthritis, or migraine - Around 3 years on opioids - Mild intensity - QoL in SF-36 with a mean of 32.9 ± 25.6 - High levels of depression and anxiety	MORE + TAU (15) - 8 weeks	TAU (15) - 8 weeks	- **Intensity**: participants in MORE reported a greater significant reduction in levels of pain. - **Emotional variables**: participants in MORE had significantly lower levels of depression and anxiety, but with increased symptoms of depression over time mainly in TAU. - **Opioid consumption**: participants in MORE had significantly less addiction and craving.
[71]/RCT	- 66% female and mean age 56.8 ± 11.7 - Primary care and pain clinics - Chronic opioid use - Country: USA	- Recurring pain stemming from chronic, non-cancer pain conditions: back pain, hip/leg/foot pain, joint pain, neck/shoulder pain, and other locations - Between 16 and 18 years of pain - Intensity of around 5 points (NRS) - Positive affect, meaning in life, savoring, self-transcendence, and opioid consumption risk - Opioid use daily or nearly daily for at least the past 90 days	MORE (50) - 8 weeks	SG (45) - 8 weeks	- **Intensity**: greater reduction of pain intensity in MORE than SG. - **Emotional variables**: MORE led to a significantly greater improvement in positive affect, meaning in life, savoring, and self-transcendence. - **Opioid consumption**: MORE showed a greater reduction in opioid consumption than SG.
[72]/RCT (pilot)	- 68% female and mean age 48 ± 14 - Primary care clinics, pain clinics, and neurology clinics - Chronic opioid use - Country: USA	- Chronic pain: lumbago, fibromyalgia, arthritis, cervical, or other - Duration not defined - Intensity of around 5.5 points (NRS) - Pain interference, opioid craving, non-reactivity to distressing thoughts and emotions, affective and somatic symptoms of stress, depression, and anger - 72% had opioid use daily or nearly daily for at least the past 90 days	MORE (57) - 8 weeks	SG (58) - 8 weeks	- **Intensity**: MORE had a significantly lower level of pain severity at post-treatment than SG. - **Emotional variables**: significantly lower levels of functional pain interference and stress at post-treatment with MORE than with SG. - **Opioid consumption**: MORE led to a significantly greater reduction in desire and opioid craving and consumption than SG. The effects of MORE were not sustained at the 3-month follow-up.
**Guided Imagery Interventions**					
[89]/RCT	- Mean age and percentage of females, respectively: - Alexander technique: 39.9 ± 7.9; 87.5% - Local heat: 40.4 ± 8.2; 100% - GI: 40.6 ± 7.8; 84% - Outpatient clinic - Country: Germany	- Chronic non-specific neck pain - Most had more than 5 years of pain - Intensity of around 60 mm (VAS scale from 0 to 100 mm) - Perceived satisfaction	Alexander technique (24) - 5 weeks	Local heat (23) - 5 weeks GI (25) - 5 weeks	- **Intensity**: significantly better results for Alexander technique compared to GI but not to the application of local heat. - **Emotional variables**: Alexander technique showed the highest satisfaction levels; GI, second highest satisfaction levels; local heat, lower satisfaction levels.
[81]/RCT	- Mean age and percentage of females, respectively: - GI: 53.65 ± 5.84; 3.4% - Control: 51.29 ± 6.51; 3.7% - Fibromyalgia - Country: Spain	- Pain related to FM - More than 3 years of pain - Intensity of around 7.7 points (VAS) - Depression	GI (30) - 4 weeks	Control (30) - 4 weeks	- **Intensity**: GI—significant reduction in pain intensity at 4 weeks (and between groups) but not at 8 weeks. Control—no significant differences in pain intensity. - **Emotional variables**: GI—significant improvement in depression at weeks 4 and 8. Control—no difference in depression over time.
[84]/RCT	- 83% female and mean age 61 - Home healthcare agency and senior citizen apartment buildings - Country: USA	- Chronic pain: arthritic, spinal disorder, fibromyalgia, hand, shoulder, vascular, post-polio syndrome, chronic leg wounds, cluster headaches, Crohn’s disease, gout, neuropathy, reflex sympathetic dystrophy, and temporomandibular disorder - Around 9.5 years of pain - Intensity between 4 and 10 (Wong–Baker FACES scale) - No emotional variables reported - 24.57% taking one or more forms of opioid analgesic	GI (21) - 4 days	Control (21) - 4 days	- **Intensity**: GI—pain became changeable and there was no recurrence of constant pain; Control—constant pain remained a strong theme.
[85]/RCT (pilot)	- Mean age and percentage of females: - GI + PMR: 72.06 ± 7.32 - Control: 74.80 ± 9.75 - 100% female - Senior citizen centers, fitness centers, churches, wellness fairs, and advertisements - Diagnosis of OA - Country: USA	- Chronic pain associated with OA - Duration not reported - Intensity of around 3.4 in pain scale from the Arthritis Impact Measures - No emotional variables reported	GI + PMR (18) - 12 weeks	Standard care (10) - 12 weeks	- **Intensity**: GI + PMR—significant reduction in pain intensity at 12 weeks when compared to control, whose members had no change in the intensity of their pain.

Legend: *n*—Number of Participants; RCT—Randomized Controlled Clinical Trial; USA—United States of America; NRS—Numeric Rating Scale; MORE—Mindfulness-Oriented Recovery Enhancement; SG—Supportive Group Psychotherapy; TAU—Treatment As Usual; HYP—Hypnosis; MM—Mindfulness Meditation; ED—Education Control; PM—Progressive Relaxation Meditation; CM—Control Meditation; cLBP/CLBP—Chronic Low Back Pain; MBCT—Mindfulness-Based Cognitive Therapy; CT—Cognitive Therapy; CBT—Cognitive Behavioral Therapy; BT—Behavioral Therapy; MBSR—Mindfulness-Based Stress Reduction; MSC—Mindfulness Self-Compassion; UC—Usual Care; UK—United Kingdom; MedExt—Meditation and Exercise to Treat Chronic Low Back Pilot Trial; ACT—Acceptance and Commitment Therapy; MRBP—Mindfulness-Based Relapse Prevention; GMV—Group Medical Visit; IMGV—Integrative Medical Group Visit; PCP—Primary Care Provider; SCI—Spinal Cord Injury; MIA—Mindfulness in Action; PE—Pain Management Psychoeducation; STM—Standardized Mindfulness; SPM—Spiritualized Mindfulness; R—Relaxation; PLP—Phantom Limb Pain; PLS—Phantom Limb Sensation; SAIPAN—SantaLucia Alleviation Intervention for Phantom in Amputees’ Neurorehabilitation; MBI—Mindfulness-Based Intervention; CTH—Chronic Tension-Type Headache; MBT—Mindfulness-Based Therapy; MBPM—Mindfulness-Based Pain Management Program; VAS—Visual Analogue Scale; GI—Guided Imagery; FM—Fibromyalgia; OA—Osteoarthritis; PMR—Progressive Muscle Relaxation.

## Data Availability

No new data were created or analyzed in this study. The original data explored in the review are stated in the article and additional inquiries can be directed to the respective contributing authors.
